# The challenges of post-COVID-19 fatigue research

**DOI:** 10.3389/fpsyg.2023.1120928

**Published:** 2023-03-27

**Authors:** Thorsten Rudroff

**Affiliations:** Department of Health and Human Physiology, University of Iowa, Iowa City, IA, United States

**Keywords:** post COVID-19, fatigue, anxiety, stress, depression, pain

As the number of confirmed COVID-19 cases exceeds 569 million globally (WHO, 2022), the amount of patients with constant symptoms is fast growing. Post-COVID-19, also referred to as long-COVID, is characterized by symptoms which are still reported 4 weeks or more after the initial COVID-19 infection and cannot be explained by an alternative diagnosis (*National Research Action Plan on Long COVID*; Department of Health Human Services, 2022). Persistent fatigue and fatigue-related symptoms such as anxiety, stress, and depression are among the main symptoms of post-COVID-19 and are independent of the severity of initial infection (hospitalized vs. non-hospitalized patients) (Townsend et al., 2020). Rudroff et al. (2020) defined post-COVID-19 fatigue as the decrease in physical and/or mental performance that results from changes in central, psychological, and/or peripheral factors.

Although the number of research studies on post-COVID-19 fatigue increases rapidly, the underlying mechanism as well as effective treatments are unclear (Joli et al., 2022). Several reasons may be accountable for this gap in knowledge. First, fatigue is a complex interplay between various physiological and psychological factors, which is derived from interoceptive feedback reflecting the homeostatic state of the body (Kluger et al., 2013; Enoka et al., 2021). In general, fatigue describes the feelings of low energy, tiredness, low motivation, and difficulty in concentration and can only be evaluated by self-report (Enoka et al., 2021). Perceived fatigability and objective fatigability do not evaluate the same underlying concept. Moreover, perceived fatigability subjectively estimates past or future work capacity, while objective fatigability measures the magnitude of change in a performance metric after a specific task (Enoka et al., 2021). Understanding the difference between fatigue, perceived fatigability, and objective fatigability is significant for the investigation of the underlying mechanisms and the improvement of efficient approaches to reduce the symptom burden of people with post-COVID-19.

Secondly, it is unclear which questionnaires provide the most meaningful assessment. Most studies have used the Fatigue Severity Scale (FSS), Fatigue Inventory (FIS) Scale, Fatigue Assessment Scale (FAS), or the Chalder Fatigue Scale. Although the 11-item Chalder Fatigue Scale seems to be the most appropriate questionnaire because it covers physical and mental fatigue, a specific post-COVID-19 fatigue questionnaire is highly warranted. In a first attempt, Naik et al. (2022) evaluated the psychometric properties of the FSS in post-COVID-19 patients, involving construct validity, data quality, and internal consistency. They found that the FSS is an appropriate tool to evaluate fatigue in these patients in future studies, including clinical trials. However, additional validation analyses (test-re-test reliability, time effect) factors such as vaccination status and COVID-19 variants should be implemented. Thirdly, various conditions, in addition to post-COVID-19, may influence fatigue and it may be difficult to determine the etiology of symptoms. For example, perceived fatigability may be exacerbated by emotional factors such as anxiety, stress, mood and motivation. More research about continuing physical symptoms after COVID-19 infection should also be concerned about mechanisms that may not be particular related to the SARS CoV-2 virus. From a clinical perspective, a thorough medical evaluation of these patients may prevent their symptoms being imprecisely attributed to COVID-19 infection and allow for identification of behavioral and cognitive mechanisms that may be aimed to lessen the symptoms. Fleischer et al. (2022) showed that the nervous system is infrequently affected in patients with post-COVID-19 syndrome and suggested that psychosomatic factors may contribute to the pathogenesis of post-COVID-19 syndrome. In this line, Matta et al. (2022) suggested that physical symptoms continuing 10–12 months after the first wave of the COVID-19 pandemic may be related more with the idea in having experienced COVID-19 rather than with an actual positive test. Importantly, previous work showed that perceived fatigability and fatigue are more prevalent than objective fatigability in post COVID-19 patients (Fietsam et al., 2022). They compared fatigue, perceived fatigue, and objective fatigability in these patients both with and without reported persistent fatigue symptoms. Interestingly, the results showed that while the subjects with fatigue symptoms did report worse levels of perceived and fatigability fatigue, they did not perform worse on an isokinetic fatigue task (objective fatigability measure) compared to subjects without fatigue symptoms. This suggests no differences in objective fatigability between the two groups. A similar pattern has been reported in people with multiple sclerosis (PwMS), with discrepancies being reported between fatigue and objective fatigability in this patient population. It has been suggested that despite comparable performance levels on a fatigue task (objective fatigability) to healthy subjects, increased perceived fatigability could be a product of increased cerebral energy demands to accomplish the same task in PwMS (DeLuca et al., 2008). To investigate this possibility, Rudroff et al. (2021) suggested that positron emission tomography (PET) imaging could be a key factor in furthering the efforts to identify the underlying mechanisms driving changes in the brain in people with post-COVID-19 fatigue symptoms. Furthermore, ^18^F-FDG-PET can be a valuable instrument to identify or rule out severe concurrent processes. Fourthly, women develop more often long-term post-COVID-19 fatigue and fatigue-related symptoms such as depression, pain, and anxiety than men (Bechmann et al., 2022; Ceban et al., 2022; Fernández-De-Las-Peñas et al., 2022). More studies, especially long-term longitudinal studies are necessary to fully comprehend the sex-related pathophysiology of fatigue and the effects of pharmacological and non-pharmacological treatments related with post-COVID-19 fatigue. Additionally, how new variants of SARS-CoV-2, such as Omicron and Delta, and vaccination influence sex differences in post-COVID-19 fatigue symptoms are crucial topics to consider. This research is essential to comprehend the natural course of post-COVID-19 fatigue and fatigue-related factors in people to utilize aimed treatment strategies (Rudroff et al., 2022). Lastly, in a perspective article by Rudroff et al. (2020), a model is introduced to describe potential factors contributing to post-COVID-19 fatigue. According to this model, conditional dependency includes the specifcity of the task, environment, and physical and mental capacity of individuals, while physiological factors include central, psychological, and peripheral aspects. Various methods are accessible to measure many of the contributing factors. Central factors can be studied *via* neuroimaging procedures such as functional magnetic resonance imaging (fMRI) and fluorodeoxyglucose -positron emission tomography (FDG-PET) (Kindred et al., 2015; Nelson et al., 2022) to measure changes in cerebral blood flow and glucose metabolism, psychological factors with neuropsychological tests (Chalah et al., 2020), and peripheral factors can be assessed by comparing variations in the electromyography signal with maximal force or power output (Proessl et al., 2018; Workman et al., 2020).

To advance knowledge in the field of post-COVID-19 fatigue symptoms, it is crucial that we understand the difference between perceived fatigability, fatigue, and objective fatigability in people and consider psychosomatic factors as possible triggers for post-COVID-19 fatigue. Moreover, neuroimaging for exploring underlying fatigue mechanisms and validated specific post-COVID-19 fatigue questionnaires should be developed.

## Author contributions

The author confirms being the sole contributor of this work and has approved it for publication.
